# Pathologic subtype-defined prognosis is dependent on both tumor stage and status of oncogenic driver mutations in lung adenocarcinoma

**DOI:** 10.18632/oncotarget.19284

**Published:** 2017-07-17

**Authors:** Yu Dong, Ying Li, Bo Jin, Jie Zhang, Jinchen Shao, Hong Peng, Shichun Tu, Baohui Han

**Affiliations:** ^1^ Department of Pulmonary Medicine, Shanghai Chest Hospital, Shanghai Jiao Tong University, Shanghai 200030, China; ^2^ Department of Pathology, Shanghai Chest Hospital, Shanghai Jiao Tong University, Shanghai 200030, China; ^3^ Department of Advocacy Section, Shanghai Chest Hospital, Shanghai Jiao Tong University, Shanghai 200030, China; ^4^ Scintillon Institute for Biomedical and Bioenergy Research, San Diego, CA 92121, USA; ^5^ Allele Biotechnology & Pharmaceuticals, Inc., San Diego, CA 92121, USA; ^6^ Shanghai Righton Biotechnology Co., Ltd, Shanghai 201403, China

**Keywords:** lung adenocarcinoma, overall survival, pathologic subtype, tumor stage, oncogenic driver mutation

## Abstract

Previous studies have shown that the prognosis of lung adenocarcinoma is associated with pathological characterization. In this study, we investigated whether pathology-based prognosis was further influenced by both tumor stage and oncogenic driver mutations. To this end, we recruited a cohort of 465 lung adenocarcinoma patients in China. These patients were classified into 6 pathology-defined subtypes i.e., lepidic-predominant adenocarcinoma (LPA), acinar-predominant adenocarcinoma (APA), papillary-predominant adenocarcinoma (PPA), micropapillary-predominant adenocarcinoma (MPA), solid-predominant adenocarcinoma (SPA), and invasive mucinous adenocarcinoma (IMA). Oncogenic mutations in *EGFR*, *KRAS*, *ALK*, *RET*, and *BRAF* genes were determined using fluorescent real-time RT-PCR. The associations of pathogenic subtype or oncogenic mutation with clinical characteristics were analyzed using Fisher’s exact tests. The interactive effects on overall survival (OS) by pathologic subtype, oncogenic mutations, and tumor stage were also determined. We have found that pathogenic subtype of lung adenocarcinoma correlated with smoking habit and tumor cell differentiation. These pathology-defined subtypes can be regrouped into 3 pathology-based prognostic groups: PPG1 (LPA), PPG2 (IMA+APA+PPA), and PPG3 (MPA+SPA) with a favorable, intermediate, and poor OS, respectively. We further demonstrated that this pathology-determined OS can be affected by both tumor stage and status of oncogenic mutations in *EGFR*, *KRAS*, *ALK*, *RET*, and *BRAF* genes. Interestingly, the presence of genetic mutations related to *ALK*, *RET* and *BRAF* had an opposite effect on OS between PPG2 (worsen) and PPG3 (improved) patients, reversing the prognostic favorability for patients within these two groups. In conclusion, prognosis of lung adenocarcinoma was defined interactively by pathologic subtype, tumor stage and oncogenic mutation.

## INTRODUCTION

Lung cancer is the leading cause of cancer death, with the highest mortality rate among all cancers in China and worldwide [1]. Lung adenocarcinoma is the most common pathological subtype of non-small cell lung cancer (NSCLC) [2]. The mutation rate of driver genes is higher in adenocarcinoma than in other subtypes of NSCLC with *EGFR*, *KRAS*, *ALK*, *RET*, and *BRAF* being those most commonly mutated [3–11]. The patients of lung adenocarcinoma with *EGFR* mutations have a better response to *EGFR* tyrosine kinase inhibitors (TKIs) than those without *EGFR* mutations, while patients that are *ALK-*positive show a better response to the TKI crizotinib, suggesting that therapeutic effectiveness can be linked to the presence of specific driver mutations [10–13].

The new classification system by the International Association for the Study of Lung Cancer/American Thoracic Society/and European Respiratory Society (IASLC/ATS/ERS) in 2011 divided lung adenocarcinoma into the following subtypes pathologically: lepidic-predominant adenocarcinoma (LPA), acinar-predominant adenocarcinoma (APA), papillary-predominant adenocarcinoma (PPA), micropapillary-predominant adenocarcinoma (MPA), solid-predominant adenocarcinoma (SPA), and invasive mucinous adenocarcinoma (IMA) [14]. Emerging evidence suggests that the characteristics defining these subtypes could be independent prognostic factors [15–20]. These studies suggest that LPA is often associated with a good prognosis, APA, PPA, and IMA are associated with an intermediate prognosis, whereas MPA and SPA are associated with the worst prognosis [15–20].

Interestingly, studies have found that the presence of driver genes, including *EGFR* and *KRAS*, are often associated with pathological subtypes in lung adenocarcinomas [18, 19, 21]. Moreover, the influence of both driver genes and pathological subtypes on lung cancer prognosis was found to correlate with TNM staging [15–20, 22, 23]. However, it is unclear whether pathological subtypes and driver genes interact to affect the prognosis. It is also unclear whether tumor stage plays a significant role affecting pathology- and/or oncogenic mutation-defined prognosis. To address these questions, we conducted a comprehensive study in a large cohort of Chinese patients with lung adenocarcinoma and determined the associations between 5 common driver genes (*EGFR, KRAS, ALK, RET, BRAF*) and pathological subtypes, as well as their combined impact on prognosis.

## RESULTS

### Patient characteristics correspond to pathological subtypes

All patients were Chinese and ranged in age from 30 to 80 years old (median age, 58.0 years). Clinical characteristics are shown in Table 1. We found PPA to be the most common subtype (226 cases, 48.6%), followed by APA (128 cases, 27.5%), IMA (41 cases, 8.8%), SPA (38 cases, 8.2%), LPA (22 cases, 4.7%), and MPA (10 cases, 2.2%). Using a Chi-square test, we identified smoking status (*P* < 0.0013) and tumor cell differentiation (*P* < 0.0001) as key clinical features associated with pathological subtyping. Compared to all adenocarcinomas, the SPA subtype correlated with sex (*P* < 0.0421), smoking history (*P* < 0.0025), and tumor cell differentiation (*P* < 0.0001) (Table 1). In addition to the SPA subtype, PPA (*P* < 0.0186) and LAP (*P* < 0.0002) subtypes also correlated with degree of tumor cell differentiation (Table 1), reflecting differences among these subtypes with distinctive molecular signatures related to their cell-of-origin (COO). These results suggested that pathological characteristics might play a significant role in determining subtype clinical manifestations.

**Table 1:** Characterization of lung adenocarcinomas (n=465) classified by the IASLC/ATS/ERS system

Clinical information	Pathological subtypes							
	Total 465 100.0%	PPA 226 48.6%	APA 128 27.5%	IMA 41 8.8%	SPA 38 8.2%	LPA 22 4.7%	MPA 10 2.2%	*P*-value
**Sex**								
Female	230	121	59	22	12	13	3	0.088
Male	235	105	69	19	26	9	7	
*P* (compared to Total)		0.331	0.5494	0.6286	0.0421	0.3934	0.3394	
**Age**								
<50	84	37	22	10	10	3	2	0.818
50–59	160	81	45	13	13	5	3	
≥60	221	108	61	18	15	14	5	
*P* (compared to Total)		0.8435	0.9706	0.6075	0.4136	0.3318	0.9568	
**Smoking history**								0.0013
Nonsmoker	312	163	79	31	16	18	5	
Smoker	153	63	49	10	22	4	5	
*P* (compared to Total)		0.1904	0.2922	0.2995	0.0025	0.1691	0.3119	
**Differentiation**								
High	178	100	42	16	1	18	1	< 0.0001
Intermediate	164	88	47	16	5	3	5	
Poor	123	38	39	9	32	1	4	
*P* (compared to Total)		0.0186	0.4817	0.7996	< 0.0001	0.0002	0.1876	
**Stage**								
I	211	107	58	18	11	14	3	0.4801
II	67	33	14	8	8	2	2	
IIIA	136	62	41	12	12	6	3	
IIIB/IV	51	24	15	3	7	0	2	
*P* (compared to Total)		0.9555	0.7537	0.7648	0.1758	0.2236	0.6957	

LPA: lepidic predominant adenocarcinoma; APA: acinar predominant adenocarcinoma; PPA: papillary predominant adenocarcinoma; MPA: micropapillary predominant adenocarcinoma; SPA: solid predominant adenocarcinoma; IMA: invasive mucinous adenocarcinoma. *P*-values were determined by Chi-square test; red font denotes statistically significant values.

### Pathologic subtypes correlate with genetic mutations

By examining oncogenic mutations in 5 driver genes, i.e. *EGFR*, *KRAS*, *ALK*, *RET*, and *BRAF*, we determined their occurring rate in each pathologic subtype and their association with these subtypes. One of these mutations was detected in 63.0% of the patients (293/465), LPA being the subtype most frequently mutated (86.4%) and MPA the least (40.0%). All detected mutations were mutually exclusive. *EGFR* mutations were most frequently detected at 49.2% (229/465), followed by *KRAS* at 8.4% (39/465), *ALK* at 3.7% (17/465), *RET* at 1.1% (5/465), and *BRAF* at 0.6% (3/465, Table 2). The distribution of these genetic mutations was significantly different among different pathological subtypes (*P* < 0.0001 by Chi-square test; Table 2). Moreover, the distribution of each individual mutated gene (except *RET*) was also significantly different among different pathological subtypes (*P* < 0.0282, 0.0025, 0.0002, 0.2271, and 0.0127 for *EGFR*, *KRAS*, *ALK*, *RET*, and *BRAF*, respectively, Table 2), suggesting that each particular type of genetic mutation (except *RET*) is likely associated with a specific type of pathological subtype.

**Table 2:** Genetic mutations and pathological subtypes

Pathologic subtypes		5-WT	*EGFR+*	*KRAS+*	*ALK+*	*RET+*	*BRAF+*	5-MT	total	*P*-value*
All subtypes	N	172	229	39	17	5	3	293	465	-
	%	37.0%	49.2%	8.4%	3.7%	1.1%	0.6%	63.0%		0.3276
LPA	N	3	15	3	1	0	0	19	22	
	%	13.6%	**68.2%**	13.6%	4.5%	0.0%	0.0%	**86.4%**		
IMA	N	12	12	10	5	0	2	29	41	< 0.0001
	%	29.3%	29.3%	**24.4%**	12.2%	0.0%	**4.9%**	70.7%		
APA	N	40	75	8	4	1	0	88	128	0.5203
	%	31.3%	58.6%	6.3%	3.1%	0.8%	0.0%	68.8%		
PPA	N	98	110	13	2	2	1	128	226	0.1923
	%	43.4%	48.7%	5.8%	0.9%	0.9%	0.4%	56.6%		
MPA	N	6	3	1	0	0	0	4	10	0.7471
	%	**60.0%**	30.0%	10.0%	0.0%	0.0%	0.0%	40.0%		
SPA	N	13	14	4	5	2	0	25	38	0.0207
	%	34.2%	36.8%	10.5%	**13.2%**	**5.3%**	0.0%	65.8%		
*P*-value^$^			0.0282	0.0025	0.0002	0.2271	0.0127	0.0136		< 0.0001#

WT: wild-type; MT: mutant; Bold font denotes the subtype with the highest mutation rate. Red color indicates statistical significance; *compared to all subtypes; ^$^compared to all negative groups; ^#^comparing all pathological subtypes and all genotypes.

Indeed, *EGFR* mutations were most common in LPA (68.2%, 15/22) and least common in IMA (29.3%, 12/41). *KRAS* mutations occurred most frequently in IMA (24.4%, 10/41) and less frequently in PPA (5.8%) and APA (6.3%). *ALK*-fusions were most common in SPA (13.2%, 5/38) and IMA (12.2%, 5/41), and least common or undetected in PPA (0.9%) and MPA (0%). *RET*-fusions were uncommon with only 5 positive cases (1.1% overall) with the highest detection rate in SPA (5.3%, 2/38). *BRAF* mutations were even less common with only 3 positive cases (0.6%) found most frequently in IMA (4.9%, 2/41). These results indicate that oncogenic mutations in specific genes occur preferably in particular lung adenocarcinoma subtypes.

Furthermore, we found that the genetic profiling defined by the presence of these 5 mutated genes was significantly different in IMA (*P* < 0.0001) and SPA (*P* < 0.0207) subtypes but not in the other subtypes when compared to that in all subtypes as a whole (Table 2). This result further supports the idea that genetic profiling is associated with pathologic characteristics.

### Prognostic determination by both pathologic subtype and tumor stage

**Figure 1:**
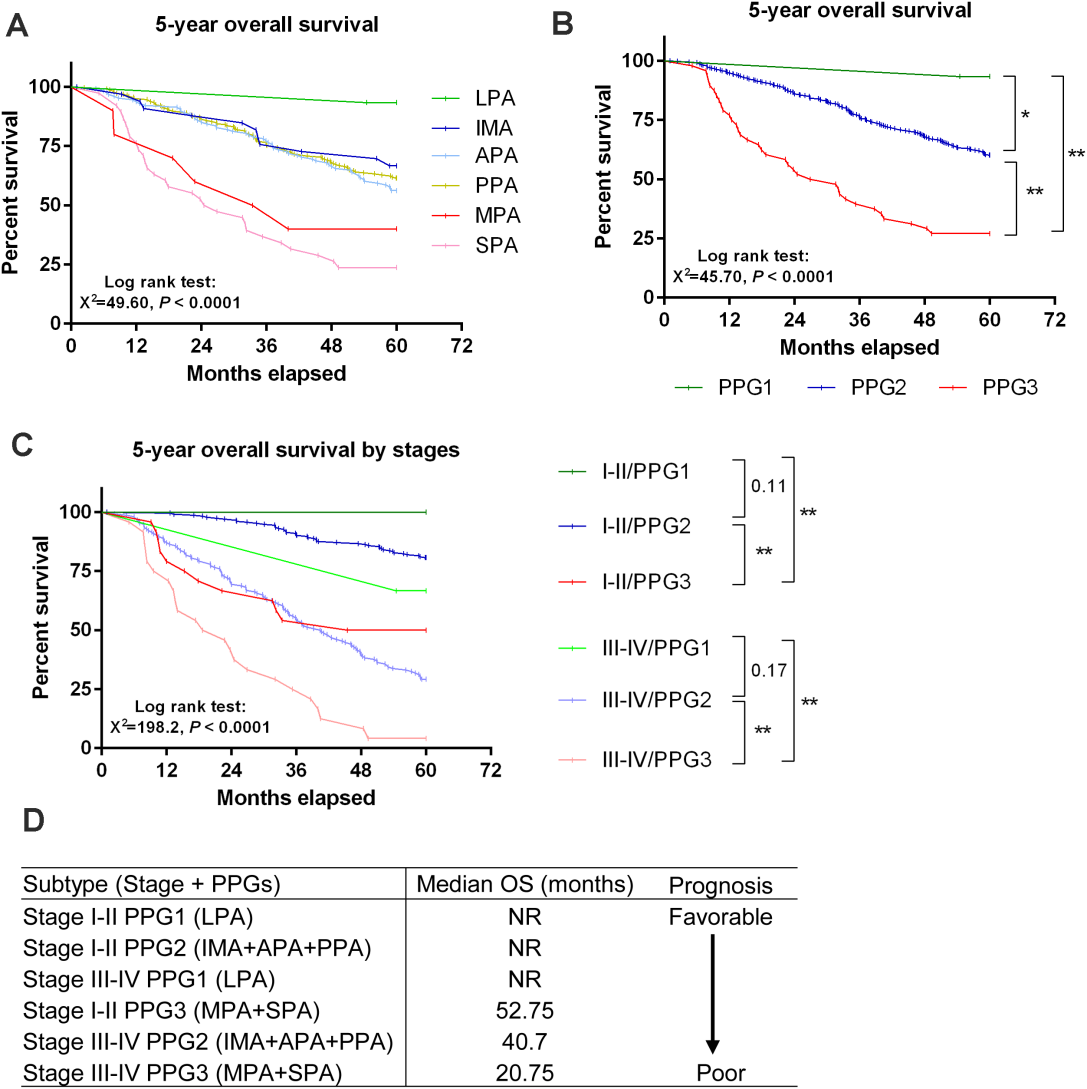
Stage-dependent prognosis was altered by pathological characteristics **(A)** 5-year OS curve by pathological subtypes. **(B)** 5-year OS curve by pathology-based prognostic group (PPG): PPG1=LPA; PPG2=IMA+APA+PPA; PPG3=MPA+SPA. **(C)** 5-year OS curve by both pathologic subtype and stage. **(D)** Schematic diagram showing the order of prognosis determined by pathologic subtype and stage. *P < 0.05 and **P < 0.01 considered as statistically significant or highly significant, respectively.

**Figure 2:**
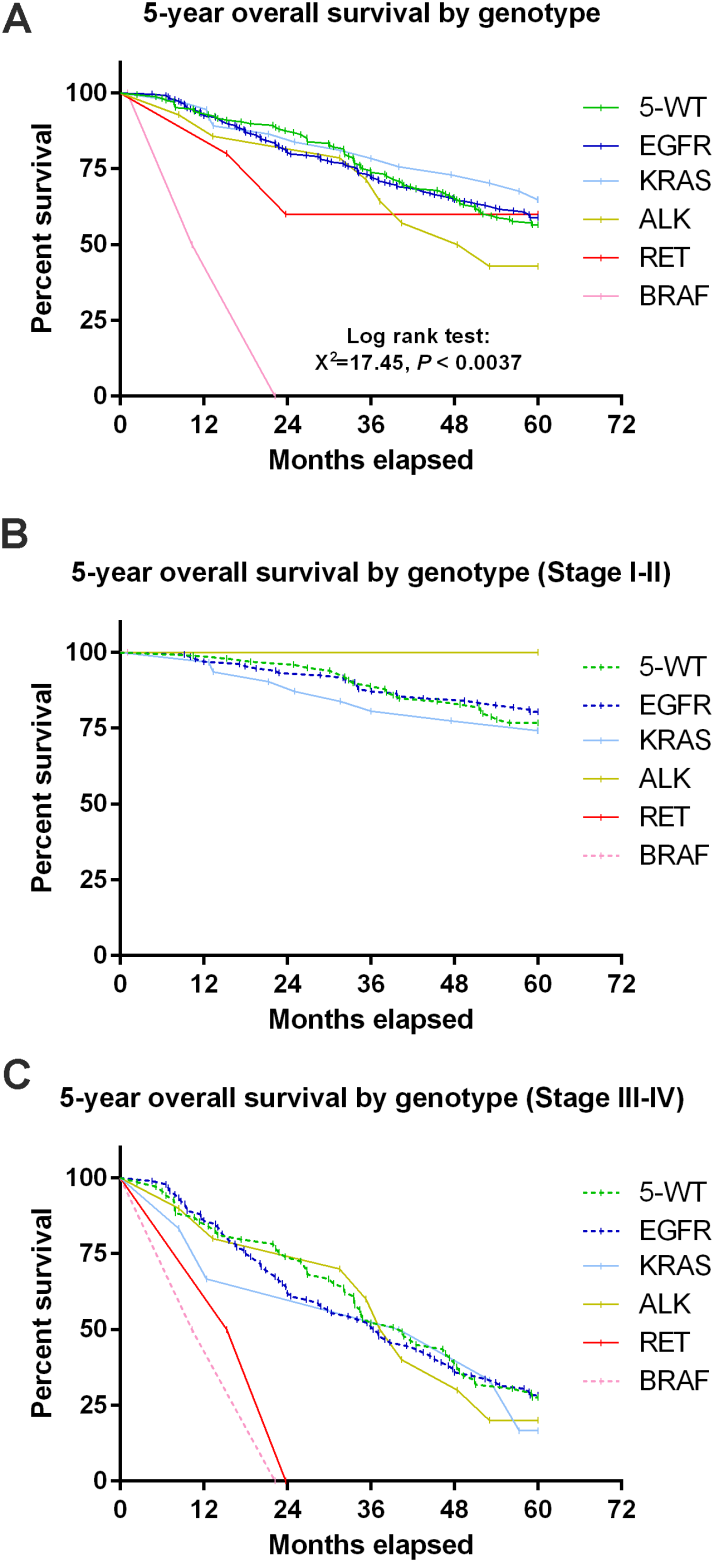
Genetic mutation-induced variation in prognosis was stage-dependent **(A)** Survival curve of 5-year OS by genotype showed that patients with *BRAF* mutations had a significantly worse OS than patients with other genotypes. **(B)** Survival curve showed that there was no significant difference in 5-year OS among stage I-II patients with different genotypes. **(C)** Patients with *BRAF* or *RET* mutations showed a significantly worse OS than patients with other genotypes at stage III/IV. *P < 0.05 considered as statistically significant.

**Figure 3:**
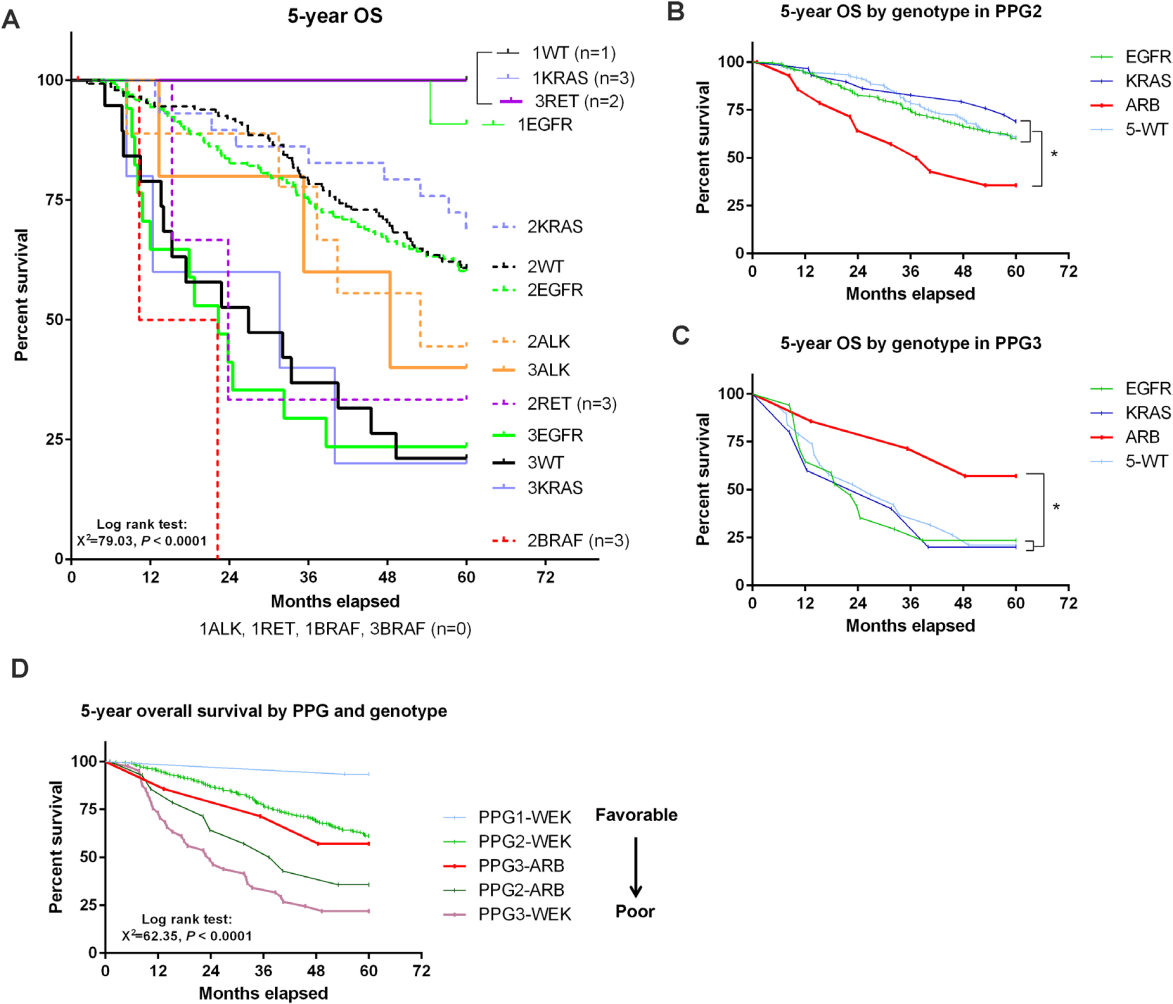
Overall survival determined by both PPG and genetic mutations **(A)** Survival curve plotted based on both PPG and genotype. 1, 2, and 3 denote PPG1, 2, and 3, respectively. **(B-C)** Survival curves by genotypes (*ALK*, *RET*, and *BRAF* were grouped together as *ARB*) in PPG2 (B) and PPG3 (C) patients. **(D)** Survival curve according to PPG and combined genotypes (ARB=*ALK*+*RET*+*BRAF* and WEK=*WT*+*EGFR*+*KRAS*). The order of prognosis from favorable to poor is indicated in the right panel. *P < 0.05 considered as statistically significant.

At the time of analysis, 206 of 451 patients (45.8%) were still alive. The median follow-up time was 68.3 months (2.4-107.9 months). We performed OS analysis (Table 3) and found a significant difference (*P* < 0.003 by Log-rank Mantel-Cox test) among patients across all subtypes harboring different genetic mutations (Table 3). We also performed a 5-year OS analysis (Figures 1-3), which showed a significant difference among different pathological subtypes (X^2^=46.13, *P* < 0.001 by Log-rank Mantel-Cox test, Figure 1A). The overall median 5-year OS was not reached in subtypes LPA, IMA, APA, and PPA, while it was 36.7 and 25.7 months in MPA and SPA subtypes, respectively. The 5-year survival rate was 57.87% for all patients and 93.33%, 67.65%, 56.25%, 61.50%, 40.00%, 23.68%, and 57.87% for subtypes LPA, IMA, APA, PPA, MPA, and SPA, respectively.

**Table 3:** OS analysis of lung adenocarcinomas with different pathologic subtypes and driver mutations

Pathologic subtypes	Median OS (months)											
	ALL n=451	EGFR+ n=224	*P*^#^	KRAS+ n=37	*P*^#^	ALK+ n=14	*P*^#^	RET+ n=5	*P*^#^	BRAF+ n=3	*P*^#^	5-WT n=168
LPA	NR	NR	0.28	NR	0.317	-	-	-	-	-	-	90.5
IMA	NR	NR	0.769	NR	0.434	72	0.671	-	-	22.2	0.002	62.5
PPA	77.5	71.4	0.388	81.5	0.681	**8.4**	0.004	15.3	<0.001	10.3	<0.001	80
APA	70.4	72.1	0.775	67.5	0.794	53	0.887	NR	0.391	-	-	65.7
MPA	33.4	NR	0.147	40	0.919	-	-	-	-	-	-	**22.8**
SPA	**24.5**	**17.9**	0.468	**12.4**	0.912	48.4	0.262	NR	0.069	-	-	26.9
All subtypes		71.4	0.977	NR	0.185	48.4	0.526	NR	0.697	10.3	<0.001	75.3
P*	0.003											

^#^Compared with 5-WT group; *compared across all subtypes and genotypes. *P* < 0.05 was considered as statistically significant (in red); NR: not reached.

Based on both 5-year OS and pathological subtypes, the patients can be divided into three distinct pathology-based prognostic groups (PPGs): PPG1: LPA (favorable), PPG2: IMA+APA+PPA (intermediate), and PPG3: MPA+SPA (poor). The 5-year OS was significantly different among the three PPGs (Table 4 and Figure 1B). The survival rate was 93.33%, 60.31%, and 27.08% for PPG1, PPG2, and PPG3, respectively. The 5-year OS was also significantly different or had a trend toward significance among the three groups at either lower (Stage I-II) or higher (III-IV) stages (Figure 1C). Interestingly, our OS analysis indicates that OS was not always better in patients at lower stages (I-II) than those at advanced stages (III-IV); e.g., PPG1 patients at advanced stages III-IV have a more favorable prognosis than PPG3 patients at lower stages I-II (Figure 1D).

**Table 4:** Five-year OS in 3 pathology-based prognostic groups

5-year OS	PPG1		PPG2	
	X^2^	*P*	X^2^	*P*
PPG1 (LPA)	-	-		
PPG2 (IMA+APA+PPA)	5.656	0.0174	-	-
PPG3 (MPA+SPA)	16.87	0.0001	37.58	0.0001

### Genetic influence on pathology-based prognosis

We next performed a 5-year OS analysis by genotypes. The 6 genetic groups (5 with mutated genes and 1 without) differed significantly in OS (*P* = 0.0037 by Log-rank Mantel-Cox test, n = 451, Figure 2A). A significant difference in OS was found between the *BRAF* (+) group (n = 3) and other mutated groups or the 5-gene negative group (*P* < 0.01, Figure 2A). Further analysis indicates that both *BRAF* and *RET* mutant patients showed a significant difference in OS for those in stage III/IV but not those in stage I/II when compared to other mutant or WT patients (*P* < 0.05 in each case, Figure 2B-2C). These results suggest that the genetic mutation-induced prognostic variation is stage-dependent.

To further determine the impact of genetic mutations on pathology- or PPG-based prognosis, we next performed a 5-year OS analysis according to 6 different genotypes in all three PPGs (Figure 3). Our results show that PPG-based OS was significantly influenced by the status of genetic mutations. We also found that mutations in *ALK*, *RET*, and *BRAF* were predominantly detected in prognostically unfavorable groups (PPG2 and 3). Based on this observation, we combined *ALK*, *RET*, and *BRAF* together (ARB) and performed individual 5-year OS analyses in PPG2 and PPG3, but not PPG1, in which the sample size was too small to give meaningful results. In PPG2, patients with *EGFR*, *KRAS*, or WT genotypes (WEK) had a significantly better prognosis than those with ARB genotypes (Figure 3B). Strikingly, in contrast to PPG2, the patients with ARB actually exhibited a better prognosis than those with WEK genotypes in PPG3 (Figure 3C). Finally we plotted a survival curve with all PPGs divided into WEK and ARB genotypes (except PPG1-ARB with no patients detected) and showed that the positivity of prognosis was in the order of PPG1-WEK > PPG2-WEK > PPG3-ARB > PPG2-ARB > PPG3-WEK (Figure 3D). PPG3-ARB patients actually showed a better prognosis than both PPG2-ARB and PPG3-WEK.

## DISCUSSION

**Figure 4:**
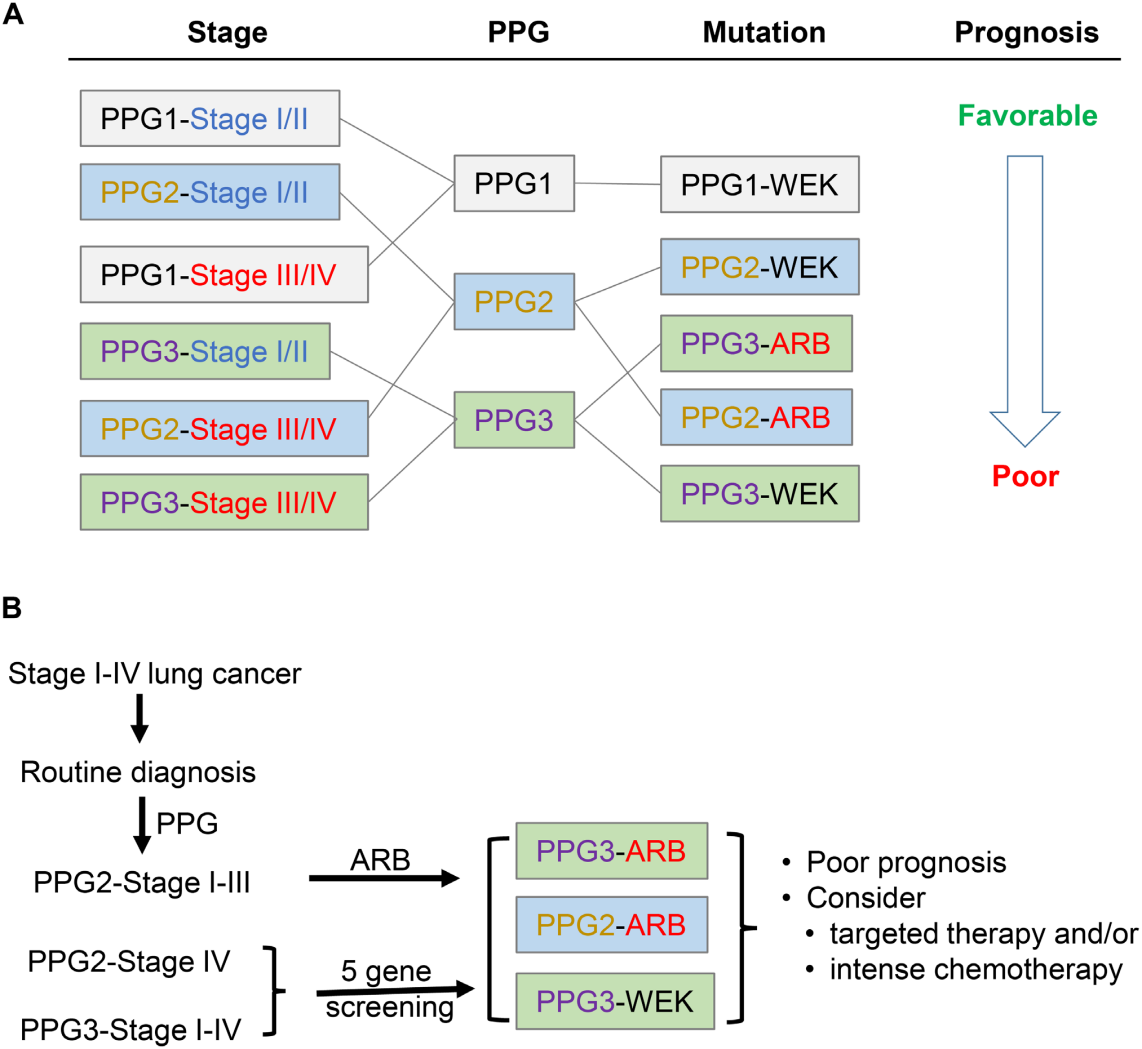
Schematic drawing indicates prognosis determined by tumor pathology, stage, and genetic mutation **(A)** Pathology-based prognosis (PPG) was significantly affected by both tumor stage and oncogenic driver mutations. **(B)** Proposed modification of tumor stage-based routine diagnostic procedures by combining pathologic subtyping and molecular characterization. According to this proposal, lung cancer patients would be subjects for PPG subtyping in addition to routine diagnostic procedures. All PPG2 and 3 patients would then be screened by either ARB or 5 gene-screening. Through these additional procedures additional subgroups of patients with poor prognosis can be isolated and considered for targeted therapy and/or intense chemotherapy.

In this study, we have integrated the effects of tumor stage, pathological characteristics, and genetic mutations in determining the prognosis of NSCLC in a cohort of 465 Chinese patients. We identified the clinical features smoking habit and tumor cell differentiation as associated with pathological subtypes. Based on patients’ prognostic values, six pathology-based subtypes can be recategorized as 3 groups that we designated as PPG1, PPG2 and PPG3 corresponding to favorable to poor OS. This pathology-defined prognosis is also dependent on both tumor stage and the status of genetic mutations in the *EGFR*, *KRAS*, *ALK*, *RET*, and *BRAF* genes. The reverse dependence of the mutation status on prognosis is also true. Specifically, the stage-dependent prognosis can be altered by pathologic characteristics, while PPG-defined favorability can be reversed by the presence of genetic mutations related to *ALK*, *RET* and *BRAF* (Figure 4A).

Our study not only verified previous findings but also illustrated new discoveries. Previous studies have shown that both pathologic subtypes and driver genes are important prognostic factors and that these two factors might be associated in lung adenocarcinomas [15–20, 22, 23]. For example, a study conducted in a Chinese population found a correlation between IMA and genetic mutations in *KRAS* and *ALK* [24]. In line with that report, we also found that *KRAS* mutations were most frequently detected in the IMA subtype, while *ALK* was most frequently detected in both IMA and SPA subtypes at nearly identical rates. Moreover, we also found that *BRAF* mutations were associated with the IMA subtype, while *EGFR* mutations were most frequently found in LPA and *RET* in SPA. Thus, mutations in 4 out of 5 driver genes were associated with either the IMA or SPA subtype, although LPA was the most frequently mutated subtype due to the high mutation rate in *EGFR*.

Further stratification has shown that the status of driver mutations added prognostic value to that of the pathologic subtype alone. It was reported that the appearance of *KRAS* and *BRAF* mutations affect the prognosis of stage IIIA patients with PPA and APA compared to a group without these mutations [24]. In our cohort, we also found that mutations in *BRAF*, but not in *KRAS*, significantly worsen OS in both PPA and IMA subtypes. The small discrepancy may reflect a difference between patients’ disease stages in the two studies. A different study has shown that although patients of stage III with APA had a better prognosis than those with MPA, MPA patients with an *EGFR* mutation had a similar prognosis to those with APA, suggesting that the presence of *EGFR* mutations significantly altered the prognosis of MPA [25]. Interestingly, we observed that there is a trend toward significance in the difference between patients with and without *EGFR* mutations in the MPA subtype. However, a contradictory result was obtained in a separate study [26], which showed that, even in the *EGFR*-mutant group, patients with an MPA tumor component still had a worse prognosis than patients without an MPA component. The discrepancy is likely due to a difference in the criteria of patient selection for performance of this comparison. Furthermore, other studies have reported that chemotherapy increased disease-free survival (DFS) or OS in MPA [27, 28]. It is therefore clear that pathology-determined prognoses can be significantly affected by genetic mutations as well as other factors.

*ALK*-fusions induce the activation of downstream canonical PI3K/AKT as well as MAPK/ERK pathways [29]. *RET* promotes cell growth through multilevel activation of STAT3 signaling [30]. Patients with *ALK* or *RET* fusion genes share many clinical characteristics, including lymphatic metastasis and subsequently worse prognoses [31–33]. Consistent with these findings, we found that the prognosis of PPA patients with *ALK* or *RET* fusion genes was significantly worse than those without (Table 3). Although targeted therapy [13, 34] and pemetrexed-based chemotherapy [35, 36] appear to be particularly effective in *ALK*- and/or *RET*-positive patients when pathologic characteristics are not considered, it remains to be determined whether these therapeutic regimes also effectively improve OS in *ALK*- or *RET*-positive PPA patients.

Our results indicate that pathological subtypes are correlated with different prognoses. Such correlations were even stronger when the six pathology-based subtypes were further re-classified into three prognostic groups PPG1 to PPG3, representing favorable to poor prognosis. Most strikingly, the prognostic pattern defined by PPG can be altered by both tumor stage (Figure 4 left) and genetic mutations (Figure 4 right). It was especially interesting that the presence of ARB (*Alk*, *RET*, or *BRAF*) mutations added an opposite prognostic value to OS when comparing patients in the PPG2 and PPG3 groups. While ARB mutations caused a worse prognosis in PPG2 patients, they triggered a more favorable prognosis in PPG3 patients. However, it should be noted that the number of patients with BRAF and RET was small, thus limiting the power of statistical interpretation of the related results.

In summary, we demonstrated that pathologic subtype has a significant impact on a patient’s prognosis with the LPA subtype having the most favorable OS and SPA the least. This impact was even more obvious when 6 pathologic subtypes were re-classified into 3 prognostic groups. However, the pathology-dependent prognosis was further influenced by both tumor stage and genetic mutations. The consideration of all three factors can provide a more accurate prognosis and result in a more precise diagnosis and treatment regimen for NSCLC (Figure 4B).

## MATERIALS AND METHODS

### Patient population and study design

This retrospective study was approved by the Institutional Review Board of our institute. From 2004 to 2012, tumor samples were collected from 465 patients with lung adenocarcinoma by surgical resection performed at Shanghai Chest Hospital, Shanghai Jiao Tong University, Shanghai, China. This cohort included 399 radical and 66 palliative surgeries. Tumor tissues were preserved as formalin-fixed, paraffin-embedded sections. Patients were excluded if (1) they previously received neoadjuvant radio-, chemo-, or targeted therapy; (2) histological samples were insufficient for genetic testing; or (3) they were diagnosed with metastatic lung adenocarcinoma. After surgery, all patients in stage IIA-IV received platinum-based combination chemotherapy every 4 weeks including Vinorelbine+Cisplatin, Gemcitabine+Cisplatin or Vinorelbine+Carboplatin, while no therapy was required for stage I patients. 87.4% of the patients receiving therapies were treated for 4 cycles, while 12.6% received only 2-3 cycles due to adverse effects. Patients with positive bronchial stump (26 cases, 5.6%) received adjuvant radiotherapy. Thirty patients received *EGFR*-TKIs after recurrence, including 23 cases with *EGFR* mutations. Clinical information including sex, age, smoking history (nonsmoker means <100 cigarettes ever), cancer stage, and tumor cell differentiation was also collected.

The patients were monitored by Chest CT and abdominal ultrasonography every three months after surgery. After release from the hospital, patients were followed through the outpatient program or phone calls every half year. The records of overall survival (OS), defined as the survival time from surgery to death or the last follow-up, was available for 451 patients, but not for the remaining 14 patients, which resulted in a follow-up rate of 97% (451/465).

### Pathology evaluation

Two clinical pathologists conducted the pathological evaluations independently. The classification of 6 lung adenocarcinoma subtypes was conducted following the 2011 IASLC/ATS/ERS guidelines [14]. The pathological staging was reassessed with the new international tumor-node-metastasis (TNM) staging system for lung cancer approved by the American Joint Committee on Cancer (AJCC, 7^th^ edition) [37]. Tumor specimens were also divided into high, intermediate, and poor groups according to the degree of tumor cell differentiation.

### Molecular analysis

Molecular analyses of *EGFR, KRAS, BRAF*, *ALK*, and *RET* were performed as described elsewhere [13, 38]. In brief, detection of genetic mutations in *EGFR, KRAS*, and *BRAF* was performed on genomic DNA, whereas *ALK and RET* fusions were determined using total RNA. Both genomic DNA and total RNA were extracted from FFPE sections. The *EGFR, KRAS*, and *BRAF* mutations were analyzed by fluorescent real-time PCR using a Human *EGFR* Mutation Detection Kit and a Human *KRAS* and *BRAF* Mutation Detection Kit (Yuanqi Bio-Pharmaceutical Co., Ltd., Shanghai, China). *ALK and RET* fusion variants were detected by multiplex One-step RT-PCR using a Human Lung Cancer Related Fusion Gene Detection Kit (Yuanqi Bio-Pharmaceutical Co.). We detected *EML4-ALK* fusion variants including EML4-E2 (V5a and 5b), EML4-E6 (V3a and 3b), EML4-E13 (V1 and 6), EML4-E14 (V4b and 7), EML4-E15 (V4a), EML4-E17 (V9), EML4-E20 (V2), and other *ALK* fusion variants including *TGF-ALK*, *KLC1-ALK*, and three *KIF5B-ALK* variants (KIF5B-E15, KIF5B-E17, and KIF5B-E24). We conducted both PCR and RT-PCR on a 7500 Real Time PCR System (ABI, Waltham, MA). We sequenced all PCR and RT-PCR products by direct sequencing to verify the presence of genetic mutations or gene fusions. The sequences of all PCR primers and sequencing probes can be found in our previously published study [38].

### Statistics

The data were analyzed using SPSS 16.0 software. Pearson’s chi-square test was used for comparisons between groups. Fisher’s exact test was used when the theoretical frequency was <5. Kaplan-Meier assays were used for the OS curves and the statistical difference was calculated by the Log-rank Mantel-Cox test. *P* < 0.05 was considered as statistically significant.

## Abbreviations

LPA: lepidic-predominant adenocarcinoma
APA: acinar-predominant adenocarcinoma
PPA: papillary-predominant adenocarcinoma
MPA: micropapillary-predominant adenocarcinoma
SPA: solid-predominant adenocarcinoma
IMA: invasive mucinous adenocarcinoma
OS: overall survival
NSCLC: non-small cell lung cancer
TKIs: tyrosine kinase inhibitors
COO: cell-of-origin
TNM: tumor-node-metastasis
AJCC: American Joint Committee on Cancer
